# Timing of anterior cruciate ligament reconstruction and preoperative pain are important predictors for postoperative kinesiophobia

**DOI:** 10.1007/s00167-019-05838-z

**Published:** 2019-12-26

**Authors:** W. W. E. S. Theunissen, M. C. van der Steen, W. Y. Liu, R. P. A. Janssen

**Affiliations:** 1 Department of Orthopaedic Surgery and Trauma, Máxima Medical Center, Ds. Th. Fliednerstraat 1, 5631 BM Eindhoven, The Netherlands; 2 Department of Orthopaedic Surgery, Catharina Hospital, Eindhoven, The Netherlands; 3 Fontys University of Applied Sciences, Eindhoven, The Netherlands; 4 Orthopaedic Biomechanics, Department of Biomedical Engineering, Eindhoven University of Technology, Eindhoven, The Netherlands

**Keywords:** Fear of movement, Fear avoidance model, Knee joint, Patient-reported outcome measures, Psychology, Tampa Scale for Kinesiophobia

## Abstract

**Purpose:**

Fear of movement (kinesiophobia) is a major limiting factor in the return to pre-injury sport level after anterior cruciate ligament reconstruction (ACLR). The aim of this study was to gain insight into the prevalence of kinesiophobia pre-ACLR, 3 months post-ACLR and 12 months post-ACLR. Furthermore, the preoperative predictability of kinesiophobia at 3 months post-ACLR was addressed.

**Methods:**

A retrospective study with data, which were prospectively collected as part of standard care, was conducted to evaluate patients who underwent ACLR between January 2017 and December 2018 in an orthopaedic outpatient clinic. Patient characteristics (age, sex, body mass index), injury-to-surgery time, preoperative pain level (KOOS pain subscale) and preoperative knee function (IKDC-2000) were used as potential predictor variables for kinesiophobia (TSK-17) at 3 months post-ACLR in linear regression analysis.

**Results:**

The number of patients with a high level of kinesiophobia (TSK > 37) reduced from 92 patients (69.2%) preoperatively to 44 patients (43.1%) 3 months postoperatively and 36 patients (30.8%) 12 months postoperatively. The prediction model, based on a multivariable regression analysis, showed a positive correlation between four predictor variables (prolonged injury-to-surgery time, high preoperative pain level, male sex and low body mass index) and a high level of kinesiophobia at 3 months postoperatively (*R*^2^ = 0.384, *p* = 0.02).

**Conclusion:**

The prevalence of kinesiophobia decreases during postoperative rehabilitation, but high kinesiophobia is still present in a large portion of the patients after ACLR. Timing of reconstruction seems to be the strongest predictor for high kinesiophobia 3 months post-ACLR. This study is the first step in the development of a screening tool to detect patients with kinesiophobia after ACLR. Identifying patients preoperatively opens the possibility to treat patients and thereby potentially increase the return to pre-injury sport level rate after ACLR.

**Level of evidence:**

III.

## Introduction

Anterior cruciate ligament reconstruction (ACLR) is performed to restore functional stability of the knee in case of an anterior cruciate ligament injury [24, 28]. Despite adequate restoration of knee function in approximately 90% of the patients [3], only 65% of the patients return to their pre-injury sport level after surgical reconstruction [5].

**Fig. 1:**
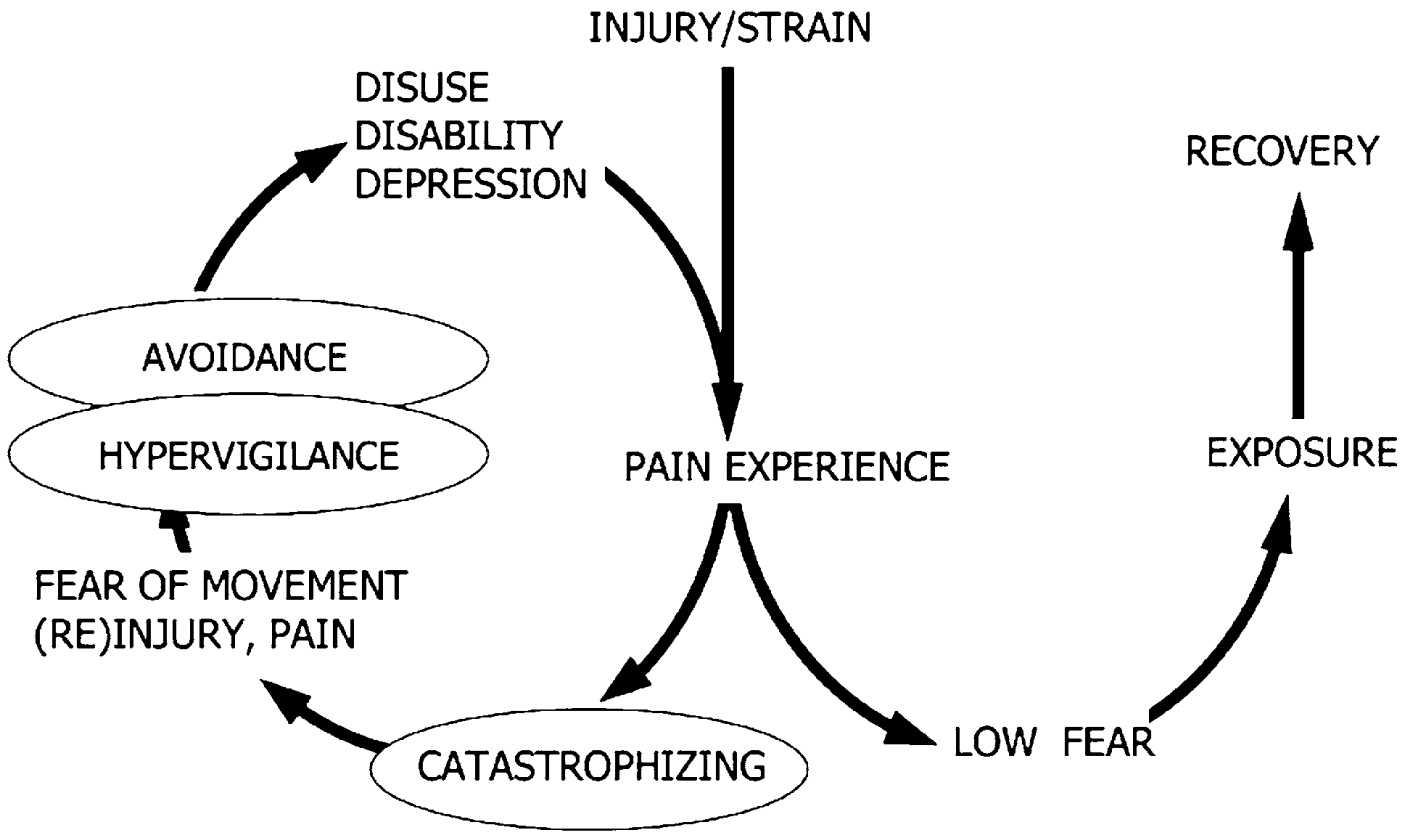
Fear avoidance model. Adapted from: Vlaeyen et al. [58]

Growing evidence suggests that psychological factors are important limiting factors for the lack of return to pre-injury sport level after ACLR [6, 7, 13]. Among these psychological factors is kinesiophobia, which is defined as ‘the fear of movement as a result of a feeling of susceptibility to pain or reinjury’ [16]. Kinesiophobia is part of the fear avoidance model [58]. This model suggests that there are two potential recovery pathways after suffering from an injury with a painful stimulus. Psychological factors such as kinesiophobia, pain catastrophizing and negative affect play a crucial role in this recovery process and when these factors turn into an irrational state of phobia, avoidance behaviour, disability and chronic pain may follow (Fig. 1) [38, 39].

Kinesiophobia has been extensively linked to ACLR. Although kinesiophobia levels seem to decrease after surgical reconstruction, high levels of kinesiophobia are still present in patients after ACLR [13, 29]. Previous research reported a prevalence of 62% at 4–8 weeks post-ACLR [48]. The current study provides new insights into the prevalence of kinesiophobia in different stages of rehabilitation after ACLR. Prevalence of kinesiophobia at 3 months postoperatively reflects the early postoperative phase in which full range of motion of the knee is restored [55]. Twelve months postoperatively reflects the final postoperative phase where the ACL graft reaches its maximum mechanical strength [31] and the regular rehabilitation process usually ends [55].

Kinesiophobia is linked to lower rates of return to sport [6, 7, 52], lower self-reported knee function [13, 18, 29] and greater risk of reinjury [6, 7, 41, 43]. Since potential interventions are most effective in the early stage of rehabilitation [60], it is of great interest to predict kinesiophobia in the early postoperative phase. Previous research has not been able to identify preoperative predictors of kinesiophobia [34]. Pain intensity [33] and the way a patient copes with pain [59] are thought to affect activity behaviour and are therefore potential predictors of kinesiophobia. However, contrary to what would be expected based on the fear avoidance model, preoperative knee pain was not identified as a predictor of kinesiophobia [34]. Another potential predictor for kinesiophobia is the duration of injury-to-surgery time (ITST). A prolonged ITST is associated with a higher level of kinesiophobia, as these patients experience a longer period of knee instability, which could lead to kinesiophobia [37, 47]. Furthermore, research in chronic low back pain patients showed that patient characteristics such as male sex and high body mass index (BMI) were related to high kinesiophobia levels [11, 56, 57].

The primary aim of the current study is to gain insight into the prevalence of kinesiophobia preoperatively, 3 months postoperatively and 12 months postoperatively in patients with a primary ACLR. The secondary aim is to analyse the preoperative predictability of kinesiophobia at 3 months postoperatively. The present study hypothesizes that kinesiophobia levels will decrease during the rehabilitation process, since graft strength [31] and knee stability [2, 51] will increase and pain levels will decrease over time [17]. It was hypothesized that patient characteristics, preoperative pain level and preoperative knee function will be potential predictor variables for kinesiophobia after ACLR.

## Materials and methods

### Study design and participants

A retrospective study with data, which were prospectively collected as part of standard care, was conducted to evaluate patients who visited the orthopaedic surgeon between January 2017 and December 2018 and underwent an ACLR at Máxima Medical Center. Inclusion criteria were primary ACLR, age ≥ 18 years and ability to complete a written survey in Dutch. Patients scheduled for revision ACLR were excluded. All surgical reconstructions were performed by two experienced orthopaedic knee surgeons using a hamstring tendon autograft. The Medical Ethical Committee at Máxima Medical Center declared that this study did not meet the criteria as stated by the Medical Research Involving Human Subjects Acts (WMO) and the local committee approved the execution of this study (METC 2018-162).

### Procedures

Patient characteristics (age, sex, BMI and injury side) and data regarding trauma mechanism, additional knee damage and ITST were extracted from the patients’ medical file and reviewed by a single researcher. Patient-reported outcome measures, collected online as part of the standard procedure to monitor the quality of care [42], were used to ascertain information on kinesiophobia (Tampa Scale for Kinesiophobia, TSK-17), pain (Knee injury and Osteoarthritis Outcome Score, KOOS pain subscale) and knee function (International Knee Documentation Committee, IKDC-2000). Patients indicated for an ACLR were asked to fill in the questionnaires preoperatively (*T*_0_) during their visit to our orthopaedic outpatient clinic. Postoperative questionnaires at three (*T*_3_) and 12 months postoperatively (*T*_12_) were linked to standard follow-up moments. These questionnaires were sent out and answered electronically or on paper. Since the TSK-17 was added to our patient-reported outcome measures in January 2018, limited follow-up data on TSK-17 scores were available. The present study chose to examine the predictability of kinesiophobia 3 months postoperatively, since potential interventions are most effective in the early stage of rehabilitation [60].

### Outcome variable

Kinesiophobia was assessed by the Dutch version of the TSK-17, a 17-item questionnaire [58]. Each item has a 4-point Likert scale with scores ranging from 1 ‘strongly disagree’ to 4 ‘strongly agree’. The total score is calculated by summing responses from all 17 items, with possible scores ranging from 17 to 68. A cutoff point of 37 was determined in previous research in chronic low back pain patients with a score > 37 indicating a high level of kinesiophobia and a score ≤ 37 a low level of kinesiophobia [58]. This cutoff point of 37 was based on the median TSK-17 score in a large group of chronic low back pain patients. The minimal clinically important difference, measured in patients with chronic low back pain, for the TSK-17 is 4 [62]. The psychometric properties of the TSK-17 are satisfactory: internal consistency (*α* = 0.76), test–retest reliability (ICC = 0.82, SEM = 3.16) and responsiveness (SRM = − 1.19) [62].

### Predictor variables

Potential predictor variables included in the multivariable analysis were determined based on literature on kinesiophobia, the fear of avoidance model and clinical expertise in light of the available data. Baseline characteristics of the study population (age, sex, BMI), injury-to-surgery time, preoperative knee pain and preoperative subjective knee function were used as predictor variables. Preoperative knee pain was assessed by the pain subscale of the Dutch version of the KOOS [19]. The KOOS consists of 42 items divided into five independent subscales: pain, symptoms, ADL, sports/recreation and quality of life. All items have five possible answers ranging from 0 ‘no problems’ to 4 ‘extreme problems’. Scores per subscale are transformed to a 0–100 scale with 0 representing extreme knee problems and 100 representing no knee problems. A total score of all five subscales combined has not been validated [45]. KOOS subscales can be used as separate outcome measures [45]. The KOOS pain subscale addresses pain intensity during nine different daily life activities [15]. The psychometric properties of the KOOS pain subscale are satisfactory: internal consistency (*α* = 0.84–0.91), test–retest reliability (ICC = 0.85–0.95) and minimal detectable change (6–6.1) [15]. Preoperative subjective knee function was assessed by the Dutch version of IKDC-2000 [30]. The IKDC-2000 comprises ten questions. Scores range from 0, representing the lowest level of function and highest level of symptoms, to 100, representing the highest level of function and lowest level of symptoms [30]. The IKDC-2000 was preferred over the KOOS as a measure of subjective knee function, as the IKDC-2000 has been reported to have a better construct validity and better responsiveness to evaluate a patient’s subjective knee function after ACLR [54].

### Statistical analysis

Statistical analysis was conducted using SPSS Statistics 25.0 (IBM Corp, Armonk, New York, United States). The prevalence of kinesiophobia at each time point was calculated by measuring the number of patients with a high kinesiophobia level (TSK > 37) divided by the total number of patients with an ACLR multiplied by 100%.

To identify preoperative predictors of kinesiophobia at 3 months postoperatively, multivariable regression analysis was used. This analysis was preceded by a comparison of the baseline characteristics of the patients who completed the TSK-17 at 3 months postoperatively between the high and low level kinesiophobia subgroup. Descriptive statistics were calculated for baseline characteristics of all patients who completed the TSK-17 at 3 months postoperatively. Unpaired sample *t* test and Chi-square test were used to compare the baseline characteristics between the subgroups. The assumption of normally distributed data was checked with the Kolmogorov–Smirnov test and a scatter plot was used to visualize the distribution. Then, multivariable regression analysis with backward selection was used to identify preoperative predictors of kinesiophobia at 3 months postoperatively. Kinesiophobia (TSK-17) at 3 months postoperatively was used as the dependent variable. Patients characteristics (age, sex, BMI), injury-to-surgery time, preoperative knee pain (KOOS pain subscale) and preoperative knee function (IKDC-2000) were used as independent variables. Backward selection starts with all potential relevant independent variables and gradually eliminates the variables that have a non-significant contribution to the multivariable model. This analysis was applied to achieve the most informative combination of predictors for kinesiophobia. Results were reported as *R*^2^, regression coefficient and *p* value. Statistical significance level for multivariable regression analysis was set at *p* < 0.05. In addition, univariable regression analysis was performed on the subset of patients who completed both the preoperative and 3 months postoperative TSK-17 to assess the predictive power of preoperative kinesiophobia.

## Results

**Fig. 2:**
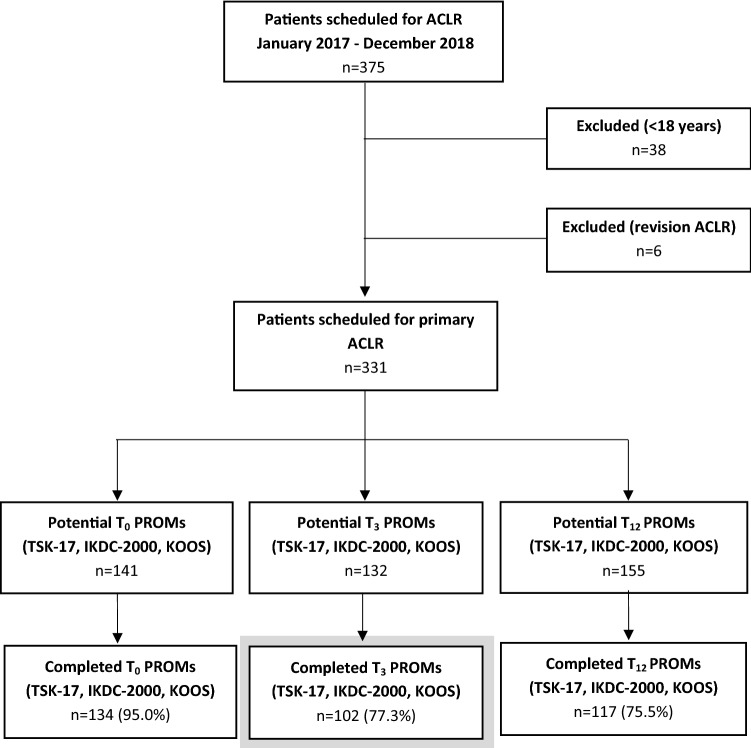
Flowchart of the study

A total of 375 patients underwent an ACLR between January 2017 and December 2018. Of these, 331 patients met the inclusion and exclusion criteria. In the end, 134 patients (95.0%) completed the TSK-17 at *T*_0_, 102 patients (77.3%) at *T*_3_ and 117 patients (75.5%) at *T*_12_. These patients were analysed to determine the prevalence of kinesiophobia (Fig. 2). The sum of this exceeds the total number of included patients, since 97 of the included patients completed the TSK-17 at two different follow-up moments (*T*_0_ and *T*_3_ or *T*_3_ and *T*_12_). Regarding our secondary aim on predictability of kinesiophobia at 3 months postoperatively, we included the 102 patients who completed the TSK-17 at *T*_3_ and used their baseline characteristics, baseline pain level (KOOS pain subscale) and baseline subjective knee function (IKDC-2000) as predictors. Both preoperative and postoperative TSK-17 was completed by 51 of these 102 patients, since the TSK-17 was integrated in our outpatient clinic from January 2018 onward.

### Prevalence of kinesiophobia

Table 1 summarizes the prevalence of kinesiophobia preoperatively, 3 months postoperatively and 12 months postoperatively in patients who underwent ACLR. At all three time points, kinesiophobia levels significantly differed between the high and low kinesiophobia subgroups: *T*_0_ 45.3 ± 5.0 vs. 33.0 ± 3.5, *T*_3_ 42.5 ± 4.9 vs. 31.9 ± 3.8 and *T*_12_ 42.0 ± 4.5 vs. 30.7 ± 6.2, *p* < 0.05). The mean difference exceeded the minimal clinical important difference of 4 points, although it should be noted that this was determined in patients with chronic low back pain.

**Table 1:** Prevalence of kinesiophobia in patients who underwent ACLR

	High kinesiophobia (TSK > 37)	
	Percentage	Number of patients
Preoperatively	69.2	92/134
3 months postoperatively	43.1	44/102
12 months postoperatively	30.8	36/117

### Potential predictors of kinesiophobia

The preoperative characteristics of the 102 patients who completed the *T*_3_ questionnaires are reported in Table 2. Patients were divided into a high kinesiophobia (TSK > 37) and low kinesiophobia (TSK ≤ 37) subgroup. Preoperative pain level and ITST were the baseline characteristics that were significantly different between the two subgroups. Other baseline characteristics (age, sex, BMI, injury side, additional knee damage, trauma mechanism) and subjective knee function were not statistically significant different between the high and low kinesiophobia subgroups.

**Table 2:** Baseline characteristics of the study population who completed *T*_3_ questionnaires

	Total (*n* = 102)	High kinesiophobia (TSK > 37) (*n* = 44)	Low kinesiophobia (TSK ≤ 37) (*n* = 58)	*p* value
Age (years)	30.5 ± 11.7	30.9 ± 12.4	30.2 ± 11.2	ns
Sex				ns
Male	59 (58%)	26 (59%)	33 (57%)	
Female	43 (42%)	18 (41%)	25 (43%)	
BMI (kg/m^2^)	24.1 ± 2.9	24.1 ± 3.4	24.0 ± 2.5	ns
ACL injury side				ns
Right	53 (52%)	22 (50%)	31 (53%)	
Left	49 (48%)	22 (50%)	27 (47%)	
Additional knee damage^a^				ns
Meniscal tear	44	19	25	
Ligament damage	27	7	20	
Bone fracture/avulsion	5	2	3	
None	36	18	18	
Trauma mechanism				ns
Contact	3 (3%)	2 (5%)	1 (2%)	
Non-contact	99 (97%)	42 (95%)	57 (98%)	
ITST (months)	5.6 ± 3.7	7.2 ± 4.7	4.3 ± 2.0	< 0.01
Pain level (KOOS pain subscale)	65.8 ± 20.9	60.9 ± 21.0	69.7 ± 20.2	0.03
Subjective knee function (IKDC)	45.2 ± 10.8	45.3 ± 9.7	45.2 ± 11.6	ns

Data are presented as mean ± standard deviation or no. (%)

*BMI* body mass index, *ACL* anterior cruciate ligament, *ITST* injury-to-surgery time, *KOOS* Knee Injury and Osteoarthritis Outcome Score, *IKDC* International Knee Documentation Committee, *TSK* Tampa Scale for Kinesiophobia

^a^Combined concomitant injuries are possible

### Predictability analysis

After applying multivariable regression analysis with backward selection, the following variables remained in the prediction model: ITST, preoperative pain level, sex and BMI. The full model, using these variables, predicts a high level of kinesiophobia 3 months postoperatively for 38.4% (*R*^2^ = 0.384, *p* = 0.02) (Table 3). The model was created with data available of 102 patients. The strongest correlation was seen with ITST (*R*^2^ = 0.20, *β* = 0.85, *p* < 0.01). A regression coefficient of 0.85 indicates that a delay of 1 month without surgery is correlated with an increase in 0.85 point on the TSK-17. Preoperative pain level was identified as the second strongest predictor of postoperative kinesiophobia (*R*^2^ = 0.11, *β* = − 0.13, *p* < 0.01). As the KOOS is inversely scored, the negative regression coefficient indicates that a 1 point increase in pain score on the KOOS pain subscale is correlated with an increase in 0.13 point on the TSK-17. Since sex and BMI were not expected to influence kinesiophobia based on the comparison between patients with high and low levels of kinesiophobia (Table 2), the present study looked into a possible confounding effect of these two variables. To this end, we investigated whether the distribution of sex was skewed for preoperative pain level and ITST. Regarding sex, preoperative pain level among women was significantly higher compared to their men counterpart (respectively, 59.8 ± 20.1 and 71.6 ± 19.2, *p* < 0.01). No sex difference in ITST was found (5.4 ± 3.0 months in women and 5.0 ± 2.3 months in men, *p* = ns). Besides, we investigated whether the distribution of BMI was skewed for preoperative pain level and ITST. For BMI, ITST appeared to be longer in the high BMI (BMI ≥ 25 kg/m^2^) compared to the low BMI (BMI < 25 kg/m^2^) subgroup (respectively, 5.8 ± 3.0 months and 4.7 ± 2.3 months; *p* = 0.04). No difference between the high BMI and low BMI subgroups in preoperative pain level was found (respectively, 65.1 ± 21.3 and 67.6 ± 19.8, *p* = ns).

**Table 3:** Multivariable prediction model of kinesiophobia at 3 months postoperatively

Dependent variable	Predictor variables	Individual *R*^2^	Combined *R*^2^	Regression coefficient (95% CI)	*p* value
Kinesiophobia	1. ITST	0.20	–	0.85 (0.54 to 1.16)	< 0.01
	2. Preoperative pain	0.11	0.31	− 0.13 (− 0.19 to − 0.08)	< 0.01
	3. Sex	0.04	0.35	3.06 (0.77 to 5.34)	0.01
	4. BMI	0.03	0.38	− 0.48 (− 0.87 to − 0.08)	0.02
	5. Preoperative knee function	0.006	0.39	− 0.06 (− 0.17 to 0.06)	ns
	6. Age	0.002	0.39	0.02 (− 0.07 to 0.12)	ns

This analysis is based on 102 patients who completed TSK-17 at *T*_3_

*ITST* injury-to-surgery time, *BMI* body mass index, sex: 0 = female, 1 = male

Univariable linear regression analysis on the subset of patients who completed both preoperative and 3 months postoperative TSK-17 revealed preoperative kinesiophobia as a strong predictor for postoperative kinesiophobia (*R*^2^ = 0.35, *β* = 0.62, *p* < 0.01).

## Discussion

The most important finding of the present study is the high prevalence of kinesiophobia pre-ACLR (69.2%), 3 months post-ACLR (43.1%) and 12 months post-ACLR (30.8%). Secondarily, this study shows that a high level of kinesiophobia at 3 months postoperatively is explained for 38.4% by a prolonged ITST, high preoperative pain level, male sex and low BMI.

This is the first study that examines the predictive value of ITST on kinesiophobia. The optimal timing of ACLR is an important clinical decision [21]. Various studies have suggested that there is an increased risk of arthrofibrosis and decreased range of motion if reconstruction is performed within 3 weeks after injury [49, 50]. More recent research contradicted this hypothesis [20]. Conversely, delayed ACLR could lead to secondary meniscal or chondral lesions [21]. The current study finds that a prolonged ITST will make patients more prone to kinesiophobia 3 months after surgical reconstruction. This result is in line with our hypothesis that patients with a longer time between injury and surgical reconstruction of the anterior cruciate ligament are more susceptible to kinesiophobia due to the experience of a prolonged time of functional knee instability [37, 47].

Following Kroska et al. [33], who stated that higher pain intensity is associated with fear avoidance behaviour, the present study suggested an association between preoperative pain and postoperative kinesiophobia. Previous research found no correlation between pain at injury time and postoperative kinesiophobia in patients who underwent an ACLR [34]. However, the patients’ ability to remember pain at injury time, 3–4 years in retrospect, could have led to recall bias in that study. Recall bias was prevented in the present study by measuring preoperative pain level in our outpatient clinic before surgical reconstruction instead of pain at injury time. Research on risk factors for kinesiophobia after total knee arthroplasty revealed that high pain intensity levels within 24 h after surgery were positively associated with high kinesiophobia levels [12]. In patients with an ACLR, a similar association was seen between high pain scores and high kinesiophobia levels in the early postoperative phase, which declined during ACLR rehabilitation [13]. Consequently, preoperative pain may play a crucial role in the development of kinesiophobia.

Since limited literature was available on preoperative predictors of kinesiophobia, the present study used backward selection to create a multivariable prediction model. Sex and BMI were included in the multivariable model, as they had a statistically significant contribution to the prediction model. Analysis of sex as a predictor for kinesiophobia revealed that male sex was associated with higher kinesiophobia levels. This result is in line with previous research in chronic low back pain patients, where men showed higher frequency of kinesiophobia (72% in men and 48% in women, *p* < 0.001) and higher level of kinesiophobia (TSK-17 score 43.4 in men and 37.7 in women, *p* < 0.001) compared to women [10]. Analysis of BMI revealed that low BMI was positively correlated with a higher kinesiophobia level. This is in contradiction to previous research in chronic low back pain patients, where a higher BMI was associated with elevated kinesiophobia levels [56, 57]. This discrepancy is explained by the limited BMI range in our sample (24.1 ± 2.9 kg/m^2^), which does not represent the BMI range of the population truthfully. The relationship between BMI and kinesiophobia needs to be further analysed in future research with a BMI range in the study population that represents the BMI range of the worldwide population.

With regard to clinical relevance, the findings of the current study will help in the development of a screening tool for kinesiophobia in patients who undergo ACLR. Since the present study is the first to examine predictors for kinesiophobia after ACLR, more research on predictor variables is needed. Based on the current study and the fear avoidance model, at least patient characteristics and information on the timing of treatment and pain experience should be part of the screening tool to identify patients who are at risk for postoperative kinesiophobia. The ultimate goal of this screening tool is early identification of patients at risk for kinesiophobia, which creates the possibility to treat patients [4, 16]. Addressing kinesiophobia early might break the vicious circle of the fear avoidance model (Fig. 1), positively affecting the rehabilitation process and possibly increasing the return to pre-injury sport level rate after ACLR. Considering the multifactorial aspect of kinesiophobia, treatment of kinesiophobia needs to be executed in a multidisciplinary rehabilitation setting [39]. Recent research showed promising results of cognitive behavioural therapy as treatment for kinesiophobia in patients after knee surgery [11, 40]. Future challenge will be to implement these therapies into the regular ACLR rehabilitation program.

Future research has to be conducted on identifying additional predictors for kinesiophobia. We have suggested, based on clinical expertise, that the number of moments of giving way could be useful, since this is related to increased knee instability. Previous research already proved the association between knee instability and high kinesiophobia levels [37, 47]. Since kinesiophobia plays a crucial role in the fear avoidance model, other psychological factors that are part of this model need to be taken into account when predicting postoperative kinesiophobia. Pain catastrophizing, which is defined as the tendency to magnify the threat value of pain stimulus [43], should be considered in future research by implementing the Pain Catastrophizing Scale. It is hypothesized that patients who tend to catastrophize their pain are more prone to kinesiophobia [38, 58]. Besides, increasing attention should be given to self-efficacy [36] as individuals with high self-efficacy seem to have a higher ability to manage challenging situations and could possibly escape the process of kinesiophobia and avoidance behaviour [8, 63].

The present study has some limitations. The retrospective study design limited the available data that could be considered as predictor variables in the multivariable model. Objective knee parameters obtained during knee examination were not documented in detail for each patient. These parameters could be useful in future research to investigate whether kinesiophobia can be objectified. In addition, no information on pre-injury sport level was available. This might have affected the ITST, since professional athletes with an anterior cruciate ligament injury commonly wish to avoid unnecessary postponement of their surgery and prefer early surgical reconstruction [20]. Furthermore, the role of preoperative kinesiophobia as predictor for postoperative kinesiophobia should be evaluated in more detail in future research. In the small subset of patients (*n* = 51) who completed the preoperative and 3 months postoperative TSK-17 questionnaire, we showed preoperative kinesiophobia as a strong predictor for postoperative kinesiophobia. The positive association between low BMI and high postoperative kinesiophobia levels found in the present study needs to be further analysed due to the limited BMI range in our sample. Since this is the first study on the predictability of kinesiophobia based on multiple preoperative factors, the results of this study have to be externally validated. External validation is essential before implementing the outcome of this study in clinical practice [9].

## Conclusions

Although the prevalence of kinesiophobia decreases during postoperative rehabilitation, high kinesiophobia is still present in a large portion of the ACLR patients. The combination of a prolonged ITST, high preoperative pain level, male sex and low BMI predicts a high level of kinesiophobia 3 months after ACLR for 38.4%. Additional parameters need to be identified to develop a screening tool for kinesiophobia to detect and treat patients during the rehabilitation process and thereby potentially increasing the return to pre-injury sport level rate after ACLR.

## Abbreviations

ACLR: Anterior cruciate ligament reconstruction
ITST: Injury-to-surgery time
TSK-17: Tampa Scale for Kinesiophobia
KOOS: Knee Injury and Osteoarthritis Outcome Score
IKDC-2000: International Knee Documentation Committee
*T*_0_: Preoperative
*T*_3_: Three months postoperative
*T*_12_: Twelve months postoperative
